# Intersubband Optical Nonlinearity of GeSn Quantum Dots under Vertical Electric Field

**DOI:** 10.3390/mi10040243

**Published:** 2019-04-12

**Authors:** Mourad Baira, Bassem Salem, Niyaz Ahamad Madhar, Bouraoui Ilahi

**Affiliations:** 1 Micro-Optoelectronic and Nanostructures Laboratory, Faculty of Sciences, University of Monastir, Monastir 5019, Tunisia; mourad.baira@isimm.rnu.tn; 2 Univ. Grenoble Alpes, CNRS, CEA/LETI Minatec, LTM, F-38000 Grenoble, France; bassem.salem@cea.fr; 3 Department of Physics and Astronomy, College of Sciences, King Saud University, Riyadh 11451, Saudi Arabia; nmadhar@ksu.edu.sa

**Keywords:** GeSn, quantum dot, electric field, intersubband nonlinear optics, absorption coefficients, refractive index changes

## Abstract

The impact of vertical electrical field on the electron related linear and 3rd order nonlinear optical properties are evaluated numerically for pyramidal GeSn quantum dots with different sizes. The electric field induced electron confining potential profile’s modification is found to alter the transition energies and the transition dipole moment, particularly for larger dot sizes. These variations strongly influence the intersubband photoabsorption coefficients and changes in the refractive index with an increasing tendency of the 3rd order nonlinear component with increasing both quantum dot (QD) size and applied electric field. The results show that intersubband optical properties of GeSn quantum dots can be successively tuned by external polarization.

## 1. Introduction

Self-assembled quantum dots have received an increasing interest during the past decades owing to their potentiality for novel optoelectronic devices [1,2]. Indeed, the strong carriers’ confinement in these nanostructures has encouraged exploring the light emission and detection in the IR [3,4,5,6] and THz regime [7,8,9] using intersubband optical transitions. A particular interest has been devoted to the study of linear and nonlinear QD intersubband optical properties [7,9,10,11,12,13,14,15,16,17] for their importance in integrated quantum photonic technologies [18]. Despite the achieved progress, efficient light source integrable with Silicon technology has, so far, represented a challenge for Si-photonic integrated circuits [19]. Recent achievement in direct band gap GeSn material has accentuated its suitability towards comparable properties to III-V materials while being compatible with complementary metal-oxide semiconductor (CMOS) technology [20,21,22,23,24,25]. Accordingly, several reports have already demonstrated the aptness of this material for optoelectronic applications, such as light emitters [25,26,27,28] and detectors [29,30,31]. Furthermore, growing experimental and theoretical research activities have been developed to explore GeSn based low dimensional structures such as quantum dots [32,33,34,35,36,37,38,39]. Indeed, different synthesis roots have been reported including, colloidal QD [33], thermal diffusion [32] and self-organization [34]. Furthermore, high Tin content GeSn QD with direct band gap transition energy has recently been reported [40]. Despite the experimental and theoretical achievement, GeSn QD are still immature and a lot of works have still to be done. Recently, we have reported on the evolution of the intersubband photoabsorption coefficients (AC) and Refractive index changes (RIC) as a function of GeSn dots size and incident radiation intensity [16]. The present work treats the effect of vertical electric field on intersubband related optical properties of pyramidal GeSn QD with different sizes for CMOS compatible nonlinear optical devices.

## 2. Theoretical Consideration

**Figure 1:**
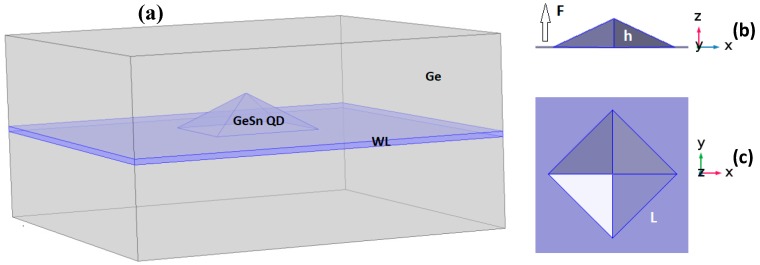
Schematic sketch of the pyramidal shaped self-assembled GeSn QD with 1 nm thick wetting layer (WL): (**a**) 3D projection of the pyramidal QD with wetting layer, (**b**) cross-sectional view (ZX) showing the QD height and the direction of the external electric field, (**c**) plane view (XY).

The self-assembled GeSn QD has been considered to have a pyramidal shape with 1nm thick wetting layer (WL) embedded in Ge matrix which is one of the frequently observed shapes for semiconducting self-assembled QD [41] as illustrated by Figure 1a. Throughout this work, we set the tin composition at 30% and a QD height to base side length’s (L) ratio of 1/3 (Figure 1b,c). 

To evaluate the QD s- and p- like electrons’ energy levels and associated wave functions in Γ-valley, single band 3D-Schrodinger equation (Equation (1)) is solved in Cartesian coordinates within the effective mass approximation by finite elements method offered by COMSOL multiphysics software (version 5.0, COMSOL Inc., Stockholm, Sweden) [42] for the strained pyramidal GeSn QD under vertical applied electric field (Figure 1).

(1) $$-\frac{\hbar^{2}}{2}\nabla \left[\frac{1}{m^{*}\left(x,y,z\right)}\nabla \emptyset \left(x,y,z\right)\right]+\left(V\left(x,y,z\right)+\mathrm{eFz}\right)\emptyset \left(x,y,z\right)=\epsilon \emptyset \left(x,y,z\right)$$

where ϵ, ∅, V and m* represent the electron energy level, envelop wave function, potential barrier and effective mass, respectively. F is the external electric field and e the elementary charge. Further details can be found elsewhere [42,43]. The calculation of the transition angular frequency associated dipole moment is mandatory to evaluate the AC and RIC. Indeed, the angular frequency dependent total intersubband optical AC (α) and RIC ($\frac{\delta n}{n_{r}}$) are given by [10,11]:

(2) $$\alpha \left(\omega \right)=\alpha^{\left(1\right)}\left(\omega \right)+\alpha^{\left(3\right)}\left(\omega ,I\right)$$

(3) $$\frac{\delta n\left(\omega \right)}{n_{r}}=\frac{\delta n^{\left(1\right)}\left(\omega \right)}{n_{r}}+\frac{\delta n^{\left(3\right)}\left(\omega \right)}{n_{r}}$$

where $\alpha^{\left(1\right)}$ and $\frac{\delta n^{\left(1\right)}}{n_{r}}$, denote the linear AC and RIC (Equations (4) and (6)). $\alpha^{\left(3\right)}$ and $\frac{\delta n^{\left(3\right)}}{n_{r}}$ represent the 3rd order nonlinear AC and RIC expressed respectively by Equations (5) and (7):

(4) $$\alpha^{\left(1\right)}\left(\omega \right)=\frac{\omega }{\hbar }\sqrt{\frac{\mu }{\varepsilon_{r}}}\frac{\sigma {\left|M_{fi}\right|}^{2}\Gamma }{\left[{\left(\omega_{fi}-\omega \right)}^{2}+\Gamma^{2}\right]}$$

(5) $$\begin{array}{ll} \alpha^{\left(3\right)}\left(\omega ,I\right)=- & \frac{\omega \sigma I}{2\varepsilon_{0}n_{r}c\hbar^{3}}\sqrt{\frac{\mu }{\varepsilon_{r}}}\frac{{\left|M_{fi}\right|}^{2}\Gamma }{{\left[{\left(\omega_{fi}-\omega \right)}^{2}+\Gamma^{2}\right]}^{2}}\\ & \times \left[4{\left|M_{fi}\right|}^{2}-\frac{{\left(M_{ff}-M_{ii}\right)}^{2}\left(3\omega_{fi}^{2}-4\omega_{fi}\omega +(\omega^{2}-\Gamma^{2})\right)}{\omega_{fi}^{2}+\Gamma^{2}}\right] \end{array}$$

(6) $$\frac{\delta n^{\left(1\right)}\left(\omega \right)}{n_{r}}=\frac{\sigma {\left|M_{fi}\right|}^{2}}{2n_{r}^{2}\varepsilon_{0}\hbar }\frac{\omega_{fi}-\omega }{\left[{\left(\omega_{fi}-\omega \right)}^{2}+\Gamma^{2}\right]}$$

(7) $$\begin{array}{ll} \frac{\delta n^{\left(3\right)}\left(\omega ,\;I\right)}{n_{r}}= & \frac{-\mu \sigma I{\left|M_{fi}\right|}^{2}}{4n_{r}^{3}\varepsilon_{0}\hbar^{3}{\left[{\left(\omega_{fi}-\omega \right)}^{2}+\Gamma^{2}\right]}^{2}}\\ & \times [4\left(\omega_{fi}-\omega \right){\left|M_{fi}\right|}^{2}\\ & -\frac{{\left(M_{ff}-M_{ii}\right)}^{2}\left\{\left(\omega_{fi}-\omega \right)\times \left[\omega_{fi}\left(\omega_{fi}-\omega \right)-\Gamma^{2}\right]-\Gamma^{2}\left(2\omega_{fi}-\omega \right)\right\}}{\omega_{fi}^{2}+\Gamma^{2}}] \end{array}$$

I is the incident in-plane polarized light intensity, σ denotes the electron density (one electron per QD) [12]. $\Gamma =10{\;\mathrm{ps}}^{-1}$ is the relaxation rate and $n_{r}$ the GeSn material’s refractive index deduced by linear interpolation [16]. $\omega_{fi}$ is the p-to-s transition frequency and $M_{fi}=\langle \emptyset_{f}|ex|\emptyset_{i}\rangle$ denotes the corresponding dipole moment for in-plane X polarized incident radiation. The subscript f and i refer to the final and initial states (QD p- and s electron states in this study). The p states are doubly degenerated (identified as px and py). A selection rule making the allowed transition to arise only from px state can be done by considering the incident radiation to be polarized along X direction [7,16,44]. 

## 3. Results and Discussion

**Figure 2:**
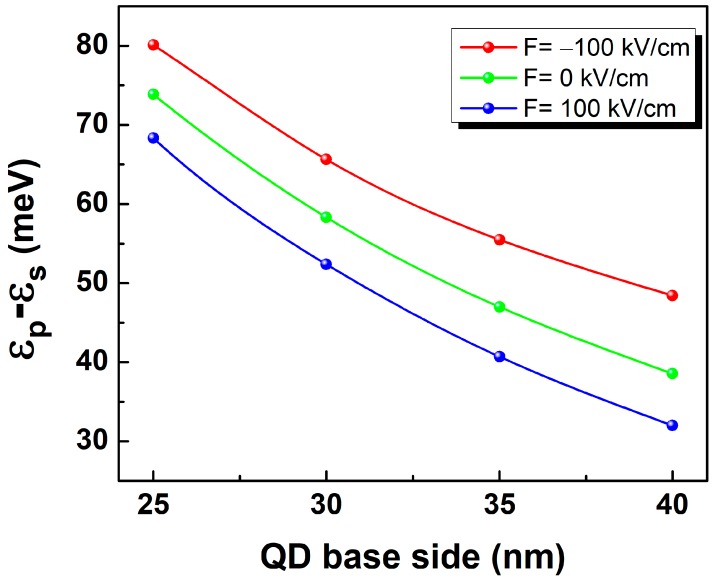
QD size dependent transition energy $\left(\epsilon_{p}-\epsilon_{s}\right)$ for F = 100kV/cm, 0 kV/cm and −100 kV/cm.

The calculation of Γ-s and -p electron energy states and associated envelop wave functions allows to evaluate the $\omega_{fi}$ and $M_{fi}$ required to study the electric field’s impact on intersubband optical properties as a function of the QD size. The transition energy $\left(\epsilon_{p}-\epsilon_{s}\right)$ is shown Figure 2 as a function of the QD size (pyramid base side) for F = 100 kV/cm, 0 kV/cm and −100kV/cm. The dot size range is delimited to L between 25 nm to 40 nm [38] warranting efficient contribution of Γ-electrons to the intersubband transition energy. 

**Figure 3:**
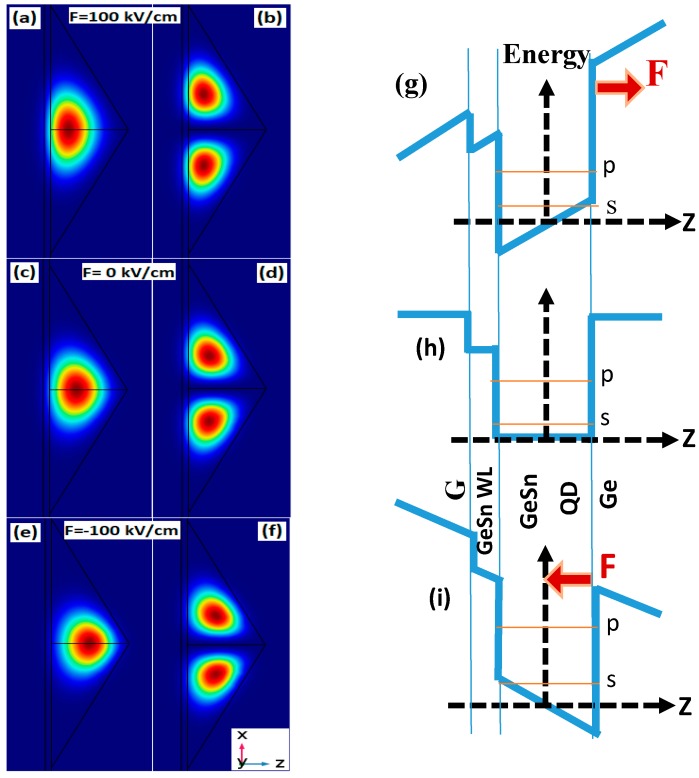
Probability density of s-state (**a**, **c** and **e**), p_x-_state (**b**, **d** and **f**) for GeSn QD with L = 40 nm as well as a simple schematic illustration of the Γ-band electron confining profile (**g**, **h** and **i**) respectively for F = 100 kV/cm, 0 kV/cm and −100 kV/cm.

In absence of electric field (F = 0 kV/cm), the intraband transition decreases from 74 meV (L = 25 nm) down to 38 meV (L = 40 nm). Applying positive electric field of 100 kV/cm enhances the transition from 6 meV for the smallest QD size up to 10 meV for the largest one. Meanwhile, the energy spacing between p and s states get rather shrank by approximately 6 meV for an external electric field of −100 kV/cm. This behavior is a direct impact of the electric field driven modification of the electron confining potential’s profile. To explain this trend, the electron probability density from s and p states (ZX plane) under an electric field of 100 kV/cm, 0 kV/cm and −100 kV/cm are shown by Figure 3 where a simplified band profile has also been provided for details. Indeed, the electric field has been found to induce a vertical shift of the electron probability density along z-axis. Its maximum gets vertically displaced towards the dot’s tip for negative electric field and towards its base for positive one [15]. Indeed, for a QD with base side length of 40 nm and a height of 13.3 nm, the maximum ground state electron probability density is located at z = 4.5 nm for unbiased QD. Under vertical electric field, the maximum is shifted upward by approximately 2.5 nm for F = −100 kV/cm and a downward vertical shift by approximately 2 nm for F = 100 kV/cm. Consequently, in the first case, the potential minimum is created near the dot tip limiting the allowed space for electron confinement (comparable environment to a QD size reduction) enhancing the separation energy between s and p states leading to the observed blueshift (Figure 2). On the other hand, the positive electric field produces a confining potential minimum at the QD base giving rise to a lowering of the confined energy states and consequent reduction of the p-to-s transition energies. 

**Figure 4:**
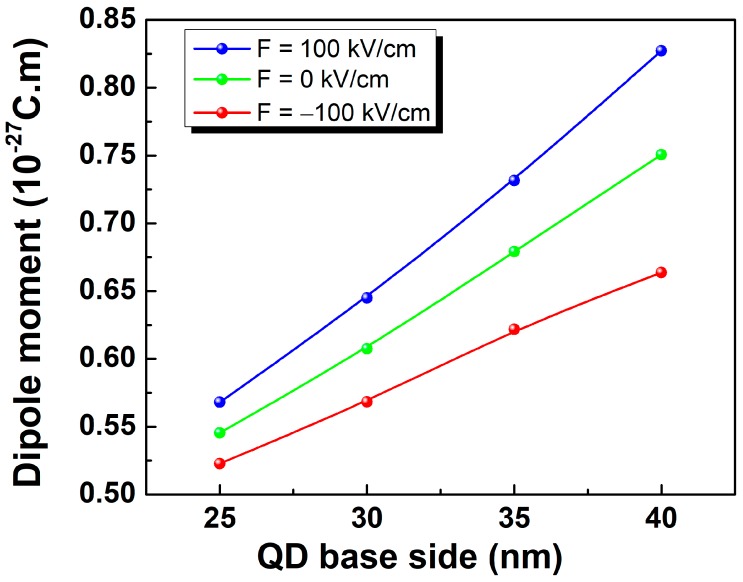
Intersubband dipole moment as a function of the pyramidal QD base side length for different values of the applied electric field.

Further information can be gained through studying the evolution of the dipole moment as a function of the dot size and electric field (Figure 4). The transition dipole moment shows an increasing trend with increasing the unbiased QD size. However, it gets progressively enhanced (decreased) with increasing the QD size upon applying 100 kV/cm (−100 kV/cm) electric field. The observed relative variation traduces a high sensitivity of larger QD sizes to the applied electric field. The obtained results show that the QD intersubband optical properties can be successively adjusted by electric polarization allowing tuning not only the intersubband emission energy but also the transition dipole moment without need for QD size variation.

**Figure 5:**
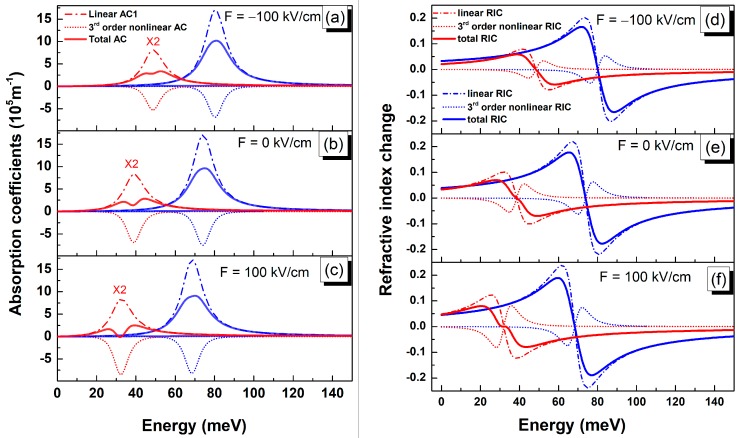
Absorption coefficients (**a**)–(**c**) and Refractive index change (**d**)–(**f**) as a function of the photon energy evaluated for F = −100 kV/cm (**a**) and (**d**), F = 0 kV/cm (**b**) and (**e**) and F = 100 kV/cm with an incident light intensity of $1\;\mathrm{MW}\cdot {\mathrm{cm}}^{-2}$. Linear contribution (dash-dot lines), 3rd order nonlinear component (dotted lines) as well as total AC and RIC (solid lines) for QD base side length: L = 25 nm (blue), L = 40 nm (red). The AC curves for L = 40 nm are multiplied by factor 2 for better visibility.

Accordingly, the impact of the dot size and electric field on the AC, RIC and the corresponding linear and third order nonlinear components are shown by Figure 5, as a function of the incident photon energy, for F = 0 kV/cm, 100 kV/cm and −100 kV/cm. The results are given for the smallest and the largest dot size to illustrate the simultaneous effect of electric field and dot size. For a given applied electric field value, the observed curves shift following the decreased transition energy with the increase of the dot size. Similarly, for a given QD size, and compared to the case where no electric field is applied, the curves get blueshifted for an electric field oriented in the negative Z direction and redshifted in the opposite case following the electric field induced intersubband transition energies shift. 

The resonance peak of the linear AC (Figure 5a–c) considerably quenches with increasing the dot size while no noticeable change is shown to occur upon the variation of the applied electric field. In the meantime, the peak’s intensity of the third-order nonlinear AC shows an increasing trend in absolute value with increasing the applied electric field for larger QD size. Consequently, the resultant total AC exhibits strong dependence on the applied electric field. When the nonlinear part of the AC becomes comparable in magnitude to the linear one, the effect of bleaching occurs inducing a splitting of the total AC into two peaks. This saturation effect observed for the unbiased larger QD size is smoothed for F = −100 kV/cm and accentuated for F = 100 kV/cm. This behavior is analogous to that perceived upon increasing the QD size and consequent variation of the absorption threshold energy [16]. 

Furthermore, the linear RIC (Figure 5 d–f) shows an overall increase with increasing the applied electric field with a pronounced sensitivity for larger dot size. Meanwhile, a similar and more accentuated variation is found to occur for the third-order nonlinear RIC affecting the total changes in the refractive index curve. The observed behavior is mainly due to the simultaneous increase of the dipole moment and decrease of the intersubband transition energy.

Our calculations clearly reveal that the intersubband optical nonlinearity can be conveniently tuned by applying an external electric field for a given QD size and incident light intensity. Accordingly, the nonlinear effects can be tuned. This investigation has been conducted on GeSn QD with the available materials parameters remain a subject to experimental validation. Nonetheless, this comprehensive study could also be useful to understand the impact of the applied electric field on the intersubband optical properties of similar QD.

## 4. Conclusions

We have evaluated the effect of applied electric field on the intersubband optical transition, dipole moment, AC, and RIC for various GeSn QD size. The transition energy and dipole moment are found to be strongly affected by the electric field-induced confining potential profile changes. Larger size QD are found to be more sensitive to the effects of applied electric field. The intersubband-related AC and RIC can be widely tuned by employing external electric field. This comprehensive study could help future realization of CMOS compatible nonlinear optical devices.
